# Cloning and characterization of the *Cerasus humilis* sucrose phosphate synthase gene *(ChSPS1)*

**DOI:** 10.1371/journal.pone.0186650

**Published:** 2017-10-16

**Authors:** Juan Wang, Junjie Du, Xiaopeng Mu, Pengfei Wang

**Affiliations:** 1 Department of Pomology, College of Horticulture, Shanxi Agricultural University, Taigu, China; 2 Institute of Pomology, Shanxi Academy of Agricultural Sciences, Taiyuan, China; National Taiwan University, TAIWAN

## Abstract

Sucrose is crucial to the growth and development of plants, and sucrose phosphate synthase (SPS) plays a key role in sucrose synthesis. To understand the genetic and molecular mechanisms of sucrose synthesis in *Cerasus humilis*, *ChSPS1*, a homologue of *SPS*, was cloned using RT-PCR. Sequence analysis showed that the open reading frame (ORF) sequence of *ChSPS1* is 3174 bp in length, encoding a predicted protein of 1057 amino acids. The predicted protein showed a high degree of sequence identity with *SPS* homologues from other species. Real-time RT-PCR analysis showed that *ChSPS1* mRNA was detected in all tissues and the transcription level was the highest in mature fruit. There is a significant positive correlation between expression of *ChSPS1* and sucrose content. Prokaryotic expression of *ChSPS1* indicated that ChSPS1 protein was expressed in *E*. *coli* and it had the SPS activity. Overexpression of *ChSPS1* in tobacco led to upregulation of enzyme activity and increased sucrose contents in transgenic plants. Real-time RT-PCR analysis showed that the expression of *ChSPS1* in transgenic tobacco was significantly higher than in wild type plants. These results suggested that *ChSPS1* plays an important role in sucrose synthesis in *Cerasus humilis*.

## Introduction

*Cerasus humilis*, commonly known as Chinese dwarf cherry, is a member of the Rosaceae, and originates in China. It is highly stress-resistant, especially to drought and cold [1]. Its fruit has high nutritional value, containing sugar, organic acid, protein, Vitamin C, and various minerals [2]. It is called ‘calcium fruit’ in China because of the significant concentration of calcium in fruit flesh (0.36%) compared to most other fruits (≤0.1%) [1,3,4]. The kernel of Chinese dwarf cherry has been used both for medicine and food for over 2000 years [5]. The fruit can be consumed fresh or be utilized in food industry, such as beverage, jam and other products [6](S1 Fig).

In higher plants, Sucrose is one of the major products of photosynthesis, and it is also the main form of translocated carbon and the main substrate for sink metabolism [7]. Most of sucrose in leaf were exported to phloem by short distance transportation, then they were unloaded into sink cells by long distance transportation; the rest of the sucrose still remain in leaf. Once unloaded into the sink cells, sucrose is disintegrated by sucrose synthase or invertase and re-synthesized rapidly by sucrose phosphate synthase [8].

Sucrose phosphate synthase (SPS) has been validated to plays a key role in carbon metabolism [9]. It is the rate-limiting enzyme in sucrose synthesis which converts fructose-6-phosphate and UDP-glucose into sucrose-6-phosphate, and then sucrose phosphatase (SPP) hydrolyzes sucrose-6-phosphate to sucrose [10–12]. It is likely that SPS regulates sucrose cycling in heterotrophic cells by providing substrates for various metabolic functions while maintaining optimal sucrose levels [13].

Numerous reports have also shown that SPS modulates development of plants. SPS activity affects sucrose accumulation during the fruit maturation stage in banana, citrus, grape, kiwifruit, pear, strawberry, tomato and watermelon [14–21]. Overexpression of a maize *SPS* gene in potato increases the rate of photosynthesis, inhibits leaf senescence, and increases yield [22]. Sucrose synthesis via *OsSPS1* is essential in pollen germination in rice [23]. In sugarcane, the *SPS* gene family is also associated with sugar-related traits, including sucrose production [24].

Although *SPS* homologues have been extensively studied in many plants, little is known about the function and expression patterns of *SPS*-like genes in *Cerasus humilis*. Differences exist in the expression patterns of *SPS* homologues in different plants. To understand the mechanism of sucrose synthesis in *Cerasus humilis*, the *SPS* homologue *ChSPS1* was cloned, and its expression pattern was analyzed by real-time RT-PCR. Its role in sucrose synthesis was investigated by verifying prokaryotic expression of *ChSPS1* and overexpressing the gene in tobacco.

## Materials and methods

### Plant materials

The *Cerasus humilis* cultivar ‘Nongda No.4’ was grown from the *Cerasus humilis* germplasm owned by Shanxi Agricultural University, Taigu, Shanxi Province, China. Fruits were harvested from June 18th to September 3rd 2016 (80d, 110d, 117d, 124d, and 131d after anthesis), from three-year-old trees and were used in real-time RT-PCR analyses. A 30-day interval was adopted between the first two developmental stages, because sugar content changes only slightly during this period [25]. Sugar content subsequently changes dramatically, so seven-day intervals were used thereafter. Roots, stems, leaves and flowers were also collected from the same trees for real-time RT-PCR. All materials were immediately frozen in liquid nitrogen after harvesting and stored at −80°C until use. The experimental protocol was approved by Shanxi Agriculture University

### Cloning the *ChSPS1* gene from *Cerasus humilis*

Total RNA was extracted from mature-stage fruits, using the TaKaRa MiniBEST Plant RNA Extraction Kit (TaKaRa, Japan), following the manufacturer’s instructions. cDNA was synthesized using a TransScript^®^ Reverse Transcriptase Kit (TransGen Biotech, Beijing, China) for real-time RT-PCR (TaKaRa, Japan) following the manufacturer’s protocol. Specific primers were designed to amplify the ORF based on the complete sequence of coding sequence (cds) from the *Prunus persica* (http://www.ncbi.nlm.nih.gov; accession umber ABV32551.1); primers were synthesized by BGI (Beijing, China). Forward and reverse primer sequences were: *ChSPS1*-F (5′-ATGGCGAGCAACGATTGGATA-3′) and *ChSPS1*-R (5′-CTACGTCTTGACAACTCCGA-3′) respectively. PCR was carried out as follows: initial denaturation at 94°C for 1 min; followed by 36 cycles of denaturation at 94°C for 30 s, annealing 59.2°C for 30 s, and elongation at 72°C for 1 min, with a final 10min extension step at 72°C. PCR products were analyzed by electrophoresis on 1% (w/v) agarose gels. PCR products were ligated into the pMD18-T cloning vector (TaKaRa, Japan), and the pMD18-*ChSPS1* was then transformed into *E*. *coli* DH5α competent cells (TransGen Biotech, Beijing, China) for sequencing by BGI.

### Gene analysis and phylogenetic tree construction

Amino acid sequences of the different *SPS* homologues were retrieved using the National Center for Biotechnology Information (NCBI Genbank) online search tool (http://www.ncbi.nlm.nih.gov/) [26]. Amino acid sequence alignments were performed with DNAMAN 6.0 software. The phylogenetic tree was constructed by MEGA 6.06, using the neighbor-joining (NJ) algorithm, based on a distance matrix calculated using the Jones-Taylor-Thornton (JTT) metric, with 1000 bootstrap replicates.

### Real-time RT-PCR analysis

In order to study the expression patterns of *ChSPS1* in *Cerasus humilis*, real-time RT-PCR analyses were conducted on root, stem, leaf, and flower, and five different developmental stages of fruit. Expression was also examined in young leaves of transgenic and wild type tobacco. Total RNA from each tissue sample was used as a template for cDNA synthesis using the PrimeScript^™^ RT reagent Kit with gDNA Eraser (TaKaRa, Japan).

Specific primers were designed within the *ChSPS1* ORF, to amplify a 131bp fragment, using Primer 5.0 software. The forward primer was 5′-GAGCGGAACAAGTGTGAATG-3′; the reverse primer was 5′-AGAACCCAGCCTTCCGTGT-3′. *Cerasus humilis ACTIN* was used as internal control, with primers 5′-ATCTGCTGGAAGGTGCTGAG-3′ and 5′-CCAAGCAGCATGAAGATCAA-3′. Tobacco *ACTIN* was used as an internal control, with primers 5′-CATTGGCGCTGAGAGATTCC-3′ and 5′-GCAGCTTCCATTCCGATCA-3′. RT-PCR was performed in a volume of 20 μl with 10 μl 2 × SYBR Premix Ex Taq (TaKaRa, Japan) 2 μl cDNA (100 ng), 0.4 μl ROX Reference Dye II (TaKaRa, Japan), 0.8 μl of each primer (10 μM), and 6 μl ddH_2_O. Each reaction was repeated three times. Amplification was carried out as follows: initial denaturation at 94°C for 1 min; followed by 40 cycles of denaturation at 94°C for 10 s, annealing at 55.5°C for 30 s, and elongation at 72°C for 1 min. Gene expression data were analyzed with ABI 7500 Software V2.3, and quantified using the comparative CT method (2^−ΔΔC*t*^). Statistical analysis was conducted by IBM SPSS statistics 21.

### Determination of soluble sugar content in *Cerasus humilis* fruit and correlation analysis

The soluble sugar (glucose, fructose, sucrose, sorbitol and total sugar) contents were determined using ultra-performance liquid chromatography (UPLC) (Waters 1525, USA) as described by YAO et al. [27]. Briefly, 5 g frozen fruit was dried into powder and moisture content was calculated. 0.5 g powder was extracted with 5 ml of acetonitrile solution (50%, v/v). After ultrasonic at 50°C with 20 min and centrifugation at 10,000 rpm with 15 min, supernatant was diluted to 10 ml with acetonitrile solution (50%, v/v). The solution was passed through SPE column and 0.22 μm filter. A sample of 15 μl was injected into the UPLC system for analysis. Acetonitrile/water (72:28, v/v) was used as the solvent at a flow rate 0.12 ml min^-1^ at 35°C. In addition, the standard samples were obtained from Sigma Chemicals Company Co. (USA). Calculation was analyzed using Breeze software. The correlation analysis between sugar accumulation and expression level of *ChSPS1* was conducted by IBM SPSS statistics 21.

### Prokaryotic expression and enzymatic activity assay

The fragment containing *ChSPS1* ORF and the correct restriction enzyme sites was amplified using *ChSPS1*-*Xba*I-F (5′-TGCTCTAGAATGGCGAGCAACGATTGG-3′) and *ChSPS1*-*Xho*I-R (5′-CCGCTCGAGCTACGTCTTGACAACTC-3′) as primers. The PCR product was ligated to pMD18-T vector. The obtained positive plasmids and prokaryotic expression vector pET28a plasmids were digested with *Xba*I and *Xho*I (TaKaRa, Japan), then ligated with T4 ligase (TaKaRa, Japan). The correct recombinant prokaryotic expression plasmid was named as pET28a-*ChSPS1* (S2 Fig), which was transformed into *E*. *coli* BL21 (DE3) competent cells (TransGen Biotech, Beijing, China).

The transformants were cultured overnight at 37°C in LB medium with kanamycin (100 mg/L). When the concentration of cell suspension reached an OD600 0.5–0.8, 1 mM isopropyl-β-D-thiogalactopyranoside (IPTG) was added to induce protein expression for 5 h at different temperatures (25, 30, 37°C). 1.5 ml bacterial cells were harvested by centrifugation at 12,000 rpm for 5 min and precipitation was resuspended in 100 μl 5×SDS loading buffer. The suspension solution was then boiled for 5–10 min. After they were centrifuged at 12,000 rpm for 5 min, 15 μl supernatant of each sample was analyzed by SDS-PAGE, which was performed in a 6% (v/v) polyacrylamide vertical slab gel with 5% (v/v) stacking gel. Proteins bands were separated clearly after stained by Coomassie brilliant blue R-250 and destained by Coomassie Blue Staining Destaining Solution.

10 ml bacteria cells with IPTG induction were collected by centrifugation at 12,000 rpm and 4°C for 5 min, then precipitation were resuspended in lysing buffer (50 mM Tris-HCl, 0.5 mM PMSF, 2 mM EDTA) for sonication. The SPS activity was measured in reaction mixture containing 100 mM HEPES-NaOH (pH 7.5), 15 mM MgCl_2_, 10 mM UDP-glucose, 10 mM fructose-6-phosphate, and crude enzyme extract was incubated for 30 min at 30°C. The reaction was terminated by adding NaOH, a coloration reaction was induced by adding HCl and resorcinol, and the reacted solution was examined using spectrophotometry at 480 nm. Statistical analysis was conducted by IBM SPSS statistics 21.

### Plant transformation

Cloned *ChSPS1* and the plant expression vector pBI121 were digested with *Xba*I and *Sma*I (TaKaRa, Japan), then ligated with T4 ligase (TaKaRa, Japan). The primers were: *ChSPS1*-*Xba*I-F (5′-TGCTCTAGAATGGCGAGCAACGATTGG-3′) and *ChSPS1*-*Sma*I-R (5′-TCCCCCGGGCTACGTCTTGACAACT-3′). The resulting plasmid (pBI121-*ChSPS1*) (S3 Fig), which also carried *gus* and the CaMV35S promoter, was transfected into *Agrobacterium tumefaciens* strain EHA105, which was then used to transform tobacco, using the leaf disc method [28]. Resistant calluses were screened out on MS medium supplemented with 100 mg/L kanamycin (kana). Transgenic plants were confirmed by gDNA extraction using the CTAB method [29] followed by PCR with *ChSPS1*-specific primers.

### GUS assay

Histochemical GUS assays were performed according to the procedure described by Mu et al. [6]. Leaves from kana-resistant and wild type lines were respectively placed in GUS-staining solution and incubated at 37°C overnight. After staining, the tissue was washed in 30% (v/v) ethanol for 15 min, then in 50% (v/v) ethanol for 30 min, and put into 100% (v/v) ethanol to remove all plant pigments.

### Determination of SPS activity and sucrose content in transgenic tobacco

The crude enzyme extract was obtained using the method described by Yang et al. [30], with some modifications. Fresh leaves (about 1.0 g) was ground up with 3mL of the following solution: 100 mM HEPES-NaOH (pH 7.5), 5 mM MgCl_2_, 1mM Na_2_EDTA, 2.5 mM DTT, 0.5% (w/v) BSA, and 10% (v/v) glycerol. After centrifugation at 12,000 rpm and 4°C for 20 min, supernatants were used as a crude enzyme extract. The assay of *ChSPS1* activity in tobacco was conducted following the method as described in *E*.*coli*. The sucrose contents of the transgenic tobacco lines were determined following the method as described in *Cerasus humilis*. Statistical analysis was conducted by IBM SPSS statistics 21.

## Results

### Cloning and sequence analysis of *ChSPS1*

RNA isolation and PCR amplification yielded a specific band on electrophoresis, of approximately 3000 bp. Sequencing of this band indicated that it was identical to the predicted sequence length. 3174 bp full-length ORF of *ChSPS1* was obtained from the fruit of *Cerasus humilis*, which encoded a 1057 amino acid protein (S1 Text). The molecular weight of the *ChSPS1* protein was 118 kDa, and its predicted isoelectric point (PI) was 6.10.

**Fig 1:**
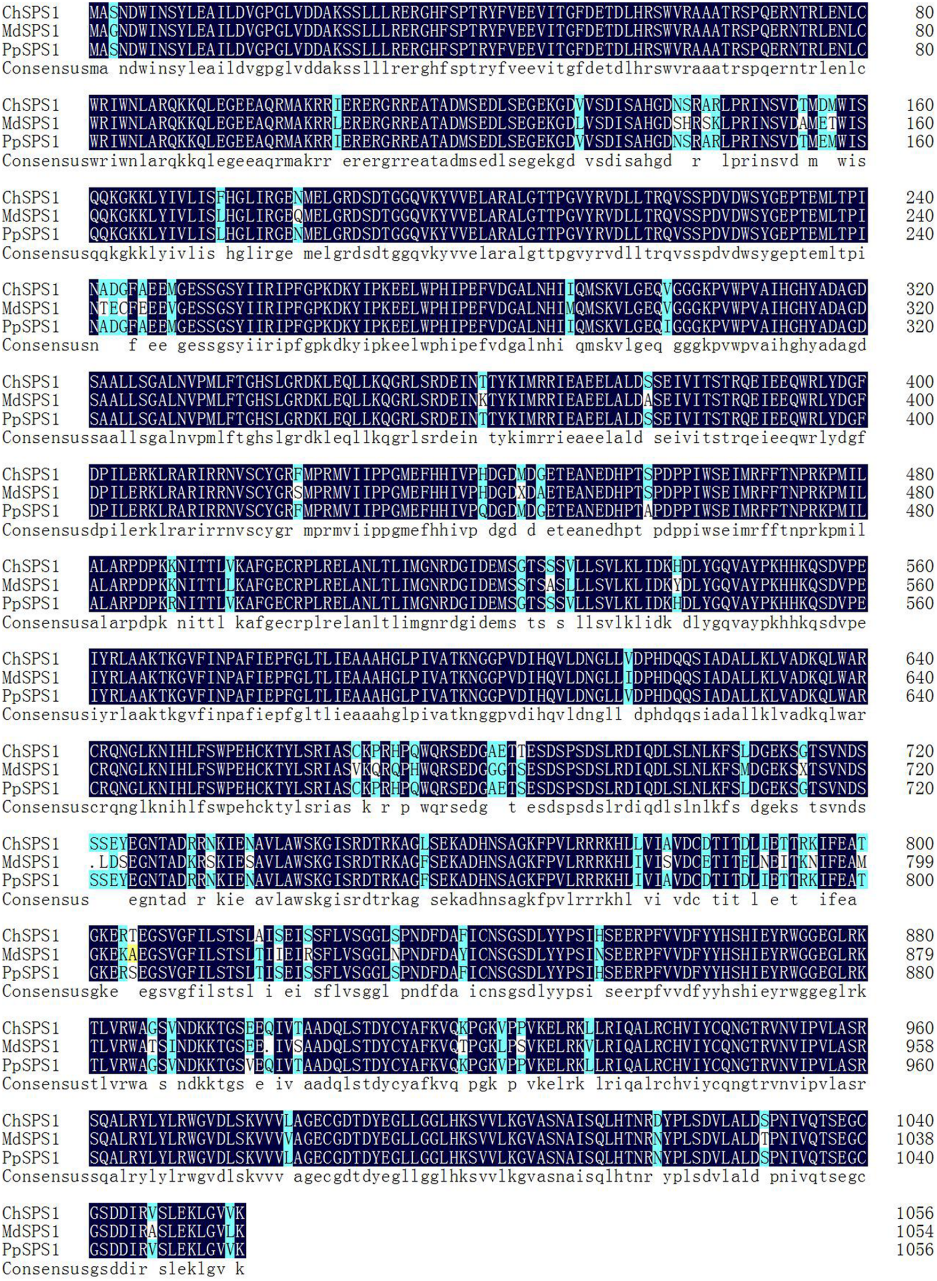
Amino acid sequence alignment of ChSPS1 from *Cerasus humilis* with SPS proteins from *Malus domestica* (GenBank accession: XP_008336979.1) and *Prunus persica* (ABV32551.1).

BLAST similarity searching showed that the deduced amino acid sequence contained significant sequence similarity to genes from other species. SPS proteins were highly conserved both in length and structure across different species. Alignment was carried out using the amino acid sequences of SPS proteins from *Prunus persica* and *Malus domestica* as references. The amino acid sequence similarities of ChSPS1 from *Cerasus humilis* with SPS proteins from *Prunus persica* and *Malus domestica* were 98.8% and 92.9%, respectively (Fig 1).

**Fig 2:**
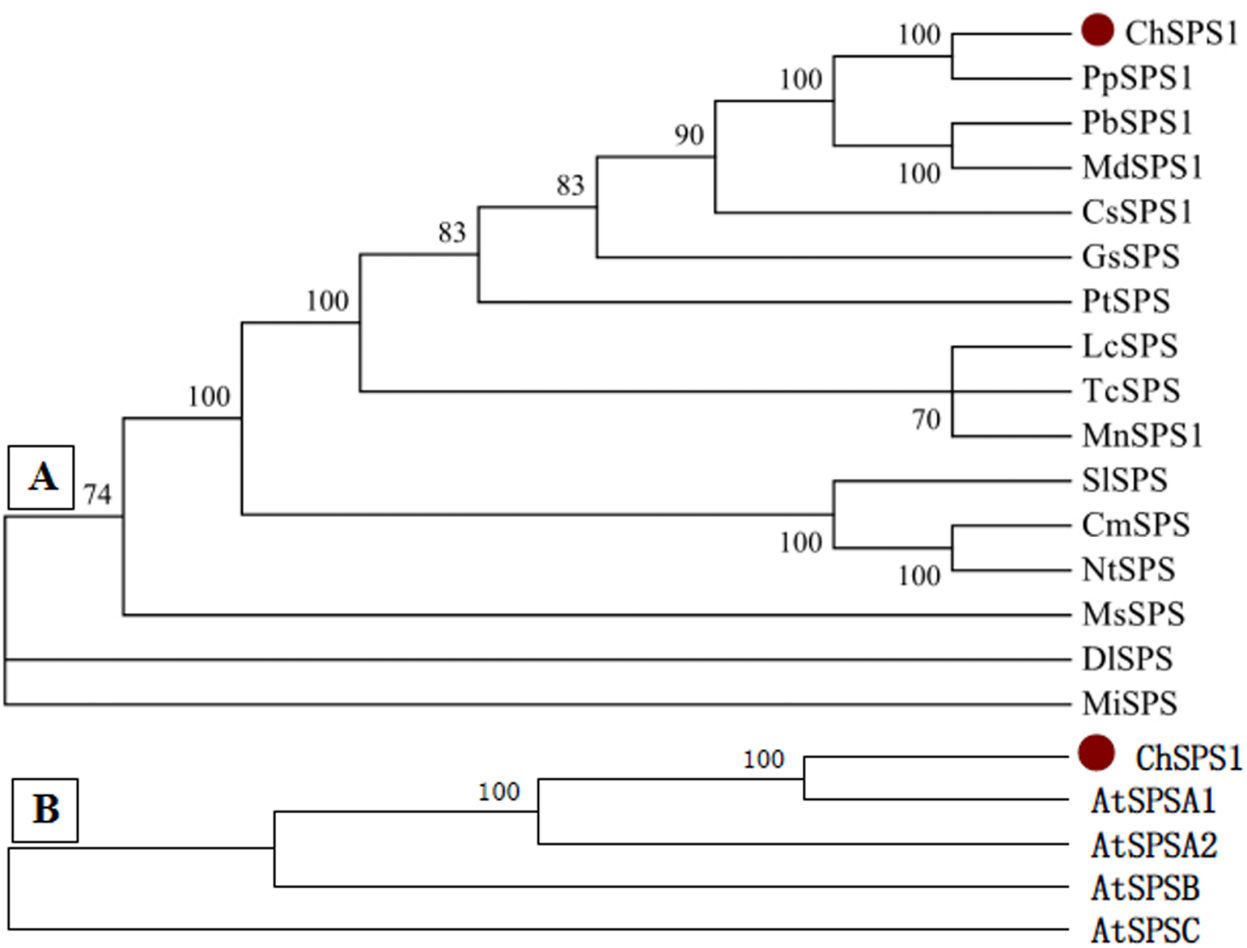
(A) Phylogenetic analysis of the amino acid sequences of *ChSPS1* with 15 other *SPS* homologues. Species names and GenBank accession numbers are: PpSPS1 (*Prunus persica*, ABV32551.1), PbSPS1 (*Pyrus bretschneideri*, XP_009363963.1), MdSPS1 (*Malus domestica*, XP_008336979.1), CsSPS1 (*Cucumis sativus*, NP_001292684.1), GsSPS (*Glycine soja*, KHN15044.1), PtSPS (*Populus trichocarpa*, XP_006389443.1), LcSPS (*Litchi chinensis*, AFP23360.1), TcSPS (*Theobroma cacao*, XP_007013574.1), MnSPS1 (*Morus notabilis*, XP_010099850.1), SlSPS (*Solanum lycopersicum*, BAB18136.1), CmSPS (*Cucumis melo*, ABC96184.1), NtSPS (*Nicotiana tabacum*, NP_001311832.1), MsSPS (*Medicago sativa*, AAK09427.2), DlSPS (*Dimocarpus longan*, AJW82919.1), and MiSPS (*Mangifera indica*, BAM68537.1). (B) Phylogenetic analysis of the amino acid sequences of *ChSPS1* with 4 *AtSPS* homologues. AtSPSA1 (At5g20280), AtSPSA2 (At5g11110), AtSPSB (At1g04920), AtSPSC (At4g10120).

To evaluate the evolutionary relationships between ChSPS1 and apparently homologous sequences, a phylogenetic tree was constructed, containing 16 species (Fig 2A). The ChSPS1 protein examined in this study (indicated by the red dot in the figure) shared a close evolutionary relationship with PpSPS1 (from *Prunus persica*), and was more distantly related to proteins from *Dimocarpus longan* and *Mangifera indica*. The results suggest that the cloned *ChSPS1* is homologous to *SPS*. The four *AtSPS* gens from *Arabidopsis thaliana* and all other known plant *SPS* genes belonged to three families—A, B and C according to Lunn et al. [12]. The phylogenetic analysis suggested that *ChSPS1* gene (indicated by the red dot in the figure) belonged to famly A (Fig 2B)

### Spatio-temporal expression pattern of *ChSPS1*

**Fig 3:**
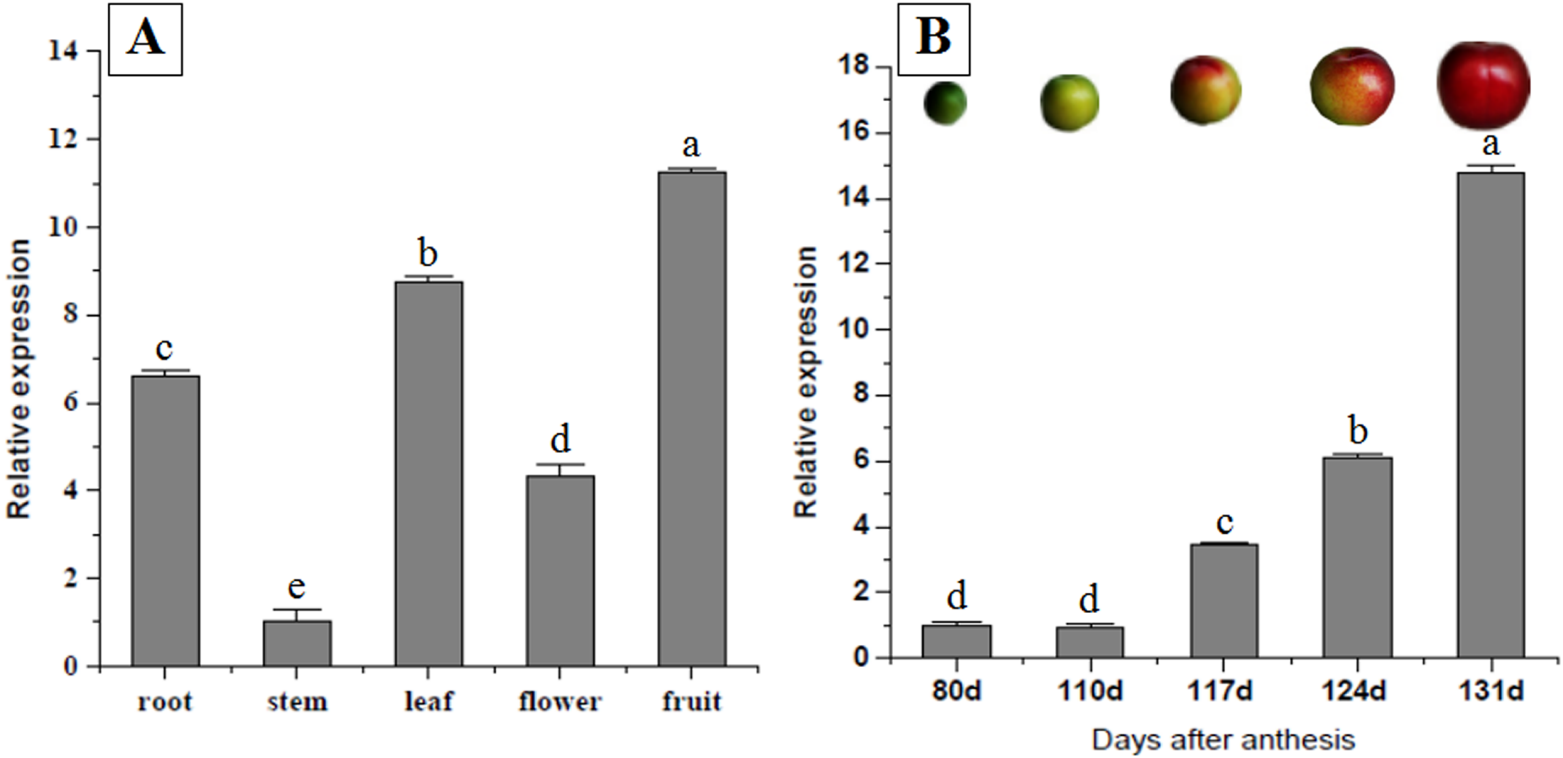
Spatio-temporal expression patterns of *ChSPS1* gene from *Cerasus humilis*. Columns with error bars indicate RQ (Relative Quantification), RQmax, RQmin respectively calculated by ABI 7500 apparatus. (A) Expression of *ChSPS1* gene at different organs (root, stem, leaf, flower and fruit); (B) Expression of *ChSPS1* gene at different developmental stages after anthesis (80d, 110d, 117d, 124d, 131d). The error bars represent standard deviation, and different lowercase letters indicate significant statistical difference at p<0.05.

Real-time RT-PCR was performed to investigate *ChSPS1* expression patterns in various tissues (root, stem, leaf, flower and fruit) and different developmental stages after anthesis of *Cerasus humilis*. The result indicated *ChSPS1* was expressed in all tissues, with the highest level of *ChSPS1* mRNA in fruit (Fig 3A). Transcription of *ChSPS1* was at its highest level during the late stage of fruit ripening (Fig 3B).

### Soluble sugar content in *Cerasus humilis* fruit and correlation analysis

The contents of soluble sugar for cultivar ‘Nongda No.4’ at five different developmental stages (80d, 110d, 117d, 124d, and 131d after anthesis) were determined. The result showed that the sucrose content, glucose content, fructose content, sorbitol content and total sugar content were all increased steadily during fruit development (Table 1). The correlation analysis suggested that the expression level of *ChSPS1* was significantly positively correlated with glucose content and sorbitol content respectively, while it was very significantly positively correlated with the total sugar and sucrose content (Table 2).

**Table 1:** The contents of soluble sugar during fruit development (mg·g^-1^FW).

DAF	Sucrose	Glucose	Fructose	Sorbitol	Total sugar
**80d**	4.177±0.436e	6.131±1.078d	15.499±0.854d	1.253±0.199d	27.061±2.197d
**110d**	5.739±1.427d	9.564±1.126c	23.252±1.056c	1.734±0.181c	40.289±3.161c
**117d**	9.426±1.174c	12.449±0.979b	25.376±1.321b	1.842±0.138c	49.093±3.181b
**124d**	16.254±1.258b	15.132±0.654a	28.884±1.963a	2.012±0.176b	62.282±3.012a
**131d**	19.882±1.187a	14.681±1.252a	28.168±1.759a	2.364±0.125a	65.095±2.252a

Different lowercase letters indicate significant statistical difference at p<0.05.

**Table 2:** The correlation analysis between soluble sugar contents and expression level of *ChSPS1*.

Gene	Correlation coefficient				
	Sucrose	Glucose	Fructose	Sorbitol	Total sugar
***ChSPS1***	0.995**	0.909*	0.857	0.949*	0.962**

* Correlation is significant at P<0.05;

**Correlation is significant at P<0.01.

### Prokaryotic expression and enzymatic activity assay

**Fig 4:**
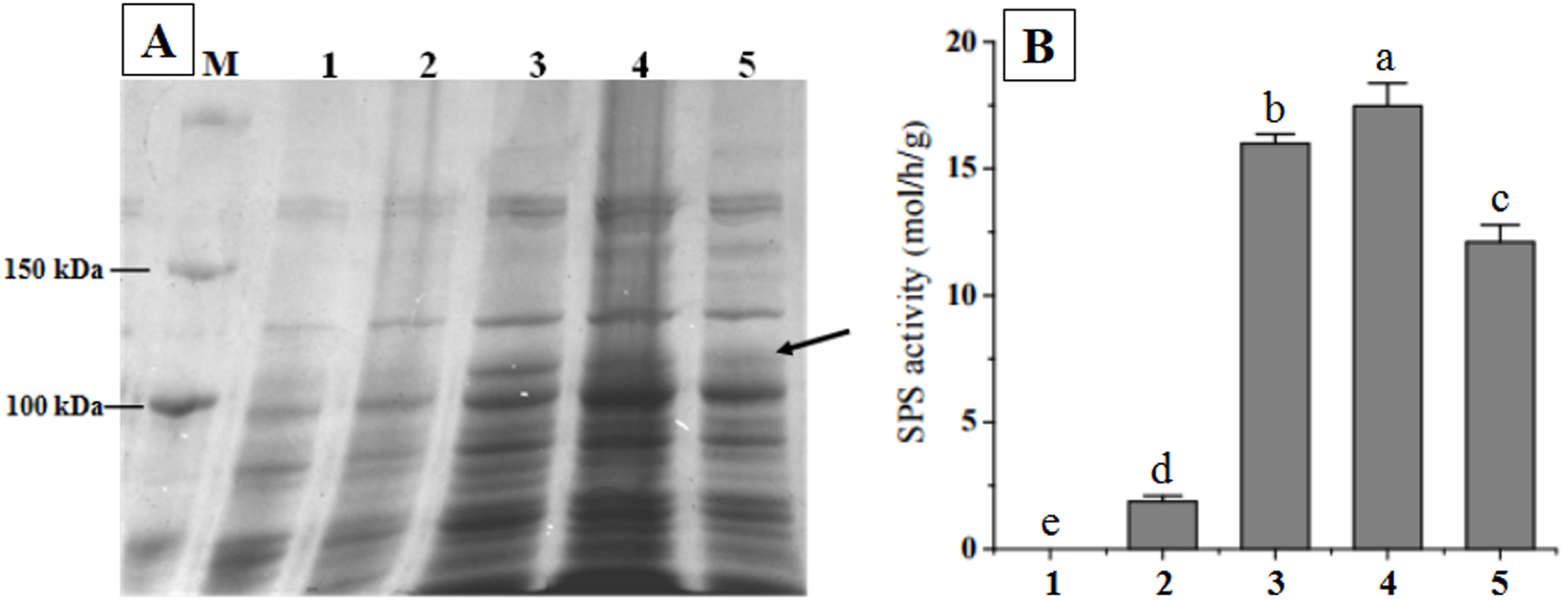
(A) SDS-PAGE analysis of the ChSPS1 protein. (B) Enzyme activity analysis of SPS in *E*. *coli*. Lane M: Protein molecular weight markers. Lane 1 and 1: Lysis of *E*. *coli*/pET28a without IPTG induction. Lane 2 and 2: Lysis of *E*. *coli*/ pET28a-*ChSPS1* without IPTG induction. Lanes 3–5 and 3–5: Lysis of *E*. *coli*/ pET28a-*ChSPS1* were induced with IPTG at 25, 30, and 37°C. The position of the objective protein is indicated by an arrow. The error bars represent standard deviation, and different lowercase letters indicate significant statistical difference at p<0.05.

The plasmids pET28a and pET28a-*ChSPS1* were transformed into *E*. *coli* BL21 (DE3) competent cell. By SDS-PAGE analysis, correct recombinant proteins with a molecular weight of approximately 118 kDa were successfully expressed (Fig 4A, Lanes 3–5), while the *E*. *coli*/pET28a control protein and *E*. *coli*/pET28a-*ChSPS1* were not found at the expected position (about 118 kDa) in the un-induced samples. The protein temperature conditions of expression were optimized to improve yield. SDS-PAGE analysis also showed that the ChSPS1 protein was optimally expressed in the transformed *E*. *coli* cell at 30°C (Fig 4A).

The SPS activity of the cells containing pET28a-*ChSPS1* was higher than that of the cells carrying empty pET28a (Fig 4B). The IPTG could significantly increase the SPS activity, probably through inducing more expression of SPS protein in the transformed *E*. *coli* cells. The result indicated that *ChSPS1* might be a functional gene encoding SPS protein, because the fusion protein had the activity of SPS.

### Overexpression of *ChSPS1* in tobacco

**Fig 5:**
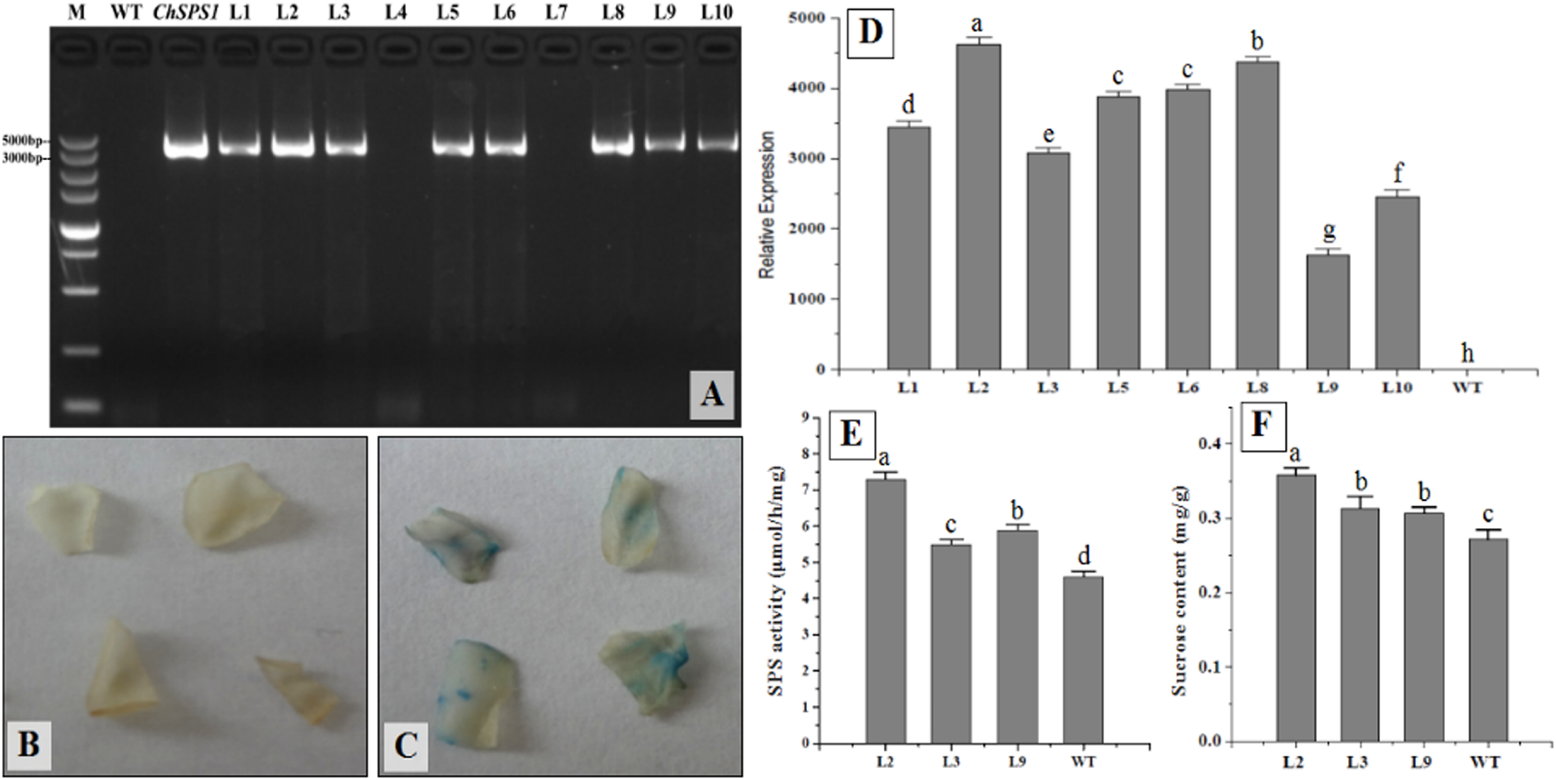
(A) PCR detection of the *ChSPS1* gene in kana-resistant shoot lines of *Cerasus humilis*. M: DNA molecular weight markers. WT: DNA from wild type tobacco. *ChSPS1*: positive control, pMD18-*ChSPS1* plasmid DNA. L1–L10: kana-resistant shoots. L4 and L7: two kana-resistant escapes. (B, C) Chemical organizational analysis of GUS. (B) wild type tobacco; (C) transgenic tobacco. (D) Expression levels of *ChSPS1* in the leaf of transgenic and wild type tobacco. (E) Enzyme activity analysis of SPS in the leaf of transgenic and wild type tobacco. (F) Sucrose content in the leaf of transgenic and wild type tobacco. The error bars represent standard deviation, and different lowercase letters indicate significant statistical difference at p<0.05.

A total of 50 Kana-resistant lines were screened on MS solid medium supplemented with 100 mg/L Kanamycin after different culture phases of tobacco (S4 Fig). Ten lines were selected at random, for gDNA extraction and real-time RT-PCR analysis with *ChSPS1*-specific primers, and GUS assays; eight lines were both PCR-positive (Fig 5A) and GUS-positive (Fig 5C), indicating that *ChSPS1* had integrated into the tobacco genome. No *gus* gene expression was detected in non-transformed control shoots (Fig 5B). Real-time RT-PCR analysis showed that the expression levels of *ChSPS1* in transgenic tobacco lines were significantly higher than those in wild type lines (Fig 5D).

SPS enzyme play an important role in plant sugar metabolism, and the overexpression of *ChSPS1* in transgenic lines may affect SPS enzyme activity and sucrose content. Among the transgenic lines, the highest *ChSPS1* expression level (L2), the middle level (L3) and the lowest level (L9) were selected to determine SPS activity and sucrose content. Both SPS activity and sucrose content of transgenic lines were higher than in the wild type line (Fig 5E and 5F).

## Discussion

Sucrose plays an important role in the plant life cycle. As the major photosynthetic product, it is essential for growth, the synthesis of biomass and as a carbon and energy source [10]. SPS is a key enzyme that regulates the sucrose synthesis pathway [31]. The *SPS* homologue *ChSPS1* was cloned from *Cerasus humilis*. The *ChSPS1* ORF was found to be 3174 bp long, encoding a predicted protein of 1057 amino acids. Sequence alignment and phylogenetic analysis showed that ChSPS1 protein has a very close evolutionary relationship with PpSPS1 (from *Prunus persica*), PbSPS1 (from *Pyrus bretschneideri*) and MdSPS1 (from *Malus domestica*). The structure of *SPS* homologues is highly conserved, and the phylogeny of these genes reflects known phylogenetic relationships [32,33]

*SPS* genes have been shown to have differential expression patterns in many plant species, including rice, alfalfa, banana, citrus, kiwifruit, peach and potato [33–39]. For example, the *SPS* gene from kiwifruit was found differentially expressed in fruits and other tissues such as roots, leaves, stems and flowers at different developmental stages [36]. The peach *PpSPS* was found highly expressed in maturing stages of fruit, while in leaves and the phloem-enriched fraction expression did not follow any particular rule during fruit development [37]. We had shown that transcription levels of *ChSPS1* were different in root, stem, leaf, flower, and fruit of *Cerasus humilis*. *ChSPS1* was expressed most strongly in fruit, with the second highest level detected in leaf. Expression increased gradually during fruit development, and there is a significant positive correlation between expression of *ChSPS1* and sucrose content, which suggests that function of *ChSPS1* may be to promote the accumulation of sucrose in fruits of *Cerasus humilis*.

Recombinant protein could be expressed using different heterologous systems including *E*. *coli*, yeast, and insect cells [40]. The *E*.*coli* based system is a typical prokaryotic expression system and has the highest expression potential [41]. In our research, *ChSPS1* protein was successfully expressed in *E*. *coli* system for the first time, and the enzyme activity assay of transformed bacterial cells showed also that *ChSPS1* encoding protein had a typical function of SPS.

Overexpression of *SPS* genes has been reported to increase or decrease sucrose contents in the transgenic plants. Alfalfa transformed with a *ZmSPS* homologue resulted in higher SPS activity and sucrose accumulation in transformants, compared to wild type alfalfa [13]. Overexpression of *ZmSPS* in tobacco not only increased the sucrose/starch ratio in transformants’ leaves, but also caused earlier flowering of tranformed plants [9]. In muskmelon, *SPS* plays an important role in regulating plant growth and determining sucrose accumulation in fruit development [42]. However, decreased sucrose contents were observed in transgenic *Arabidopsis thaliana* plants overexpressing cyanobacterial *SPS* [43]. In this study, *ChSPS1* from *Cerasus humilis* was overexpressed in tobacco and high levels of *ChSPS1* transcripts were detected in transgenic plants. *ChSPS1* overexpression led to increased SPS activities and sucrose contents in transgenic tobacco leaves which coincided with the increased SPS activity in *E*.*coli* from prokaryotic expression assay. These results showed that high levels of *ChSPS1* expression can promote sucrose accumulation either in transgenic plants or in procaryotic organism.

Hitherto, little attention has been paid to molecular biological studies of sugar accumulation in the fruits of *Cerasus humilis*. Here we have cloned and expressed *ChSPS1* protein in *E*.*coli*, meanwhile, we also transfected *ChSPS1* into tobacco and demonstrated its involvement in sucrose synthesis. These results provide a step toward better understanding of the molecular mechanisms of sugar metabolism in *Cerasus humilis*, futhermore faciliate the molecular breeding of this species.

## Supporting information

S1 Fig The phenotypic characteristic of fruits for *Cerasus humilis* cultivar ‘Nongda No.4’. (TIF)

S2 Fig Identification of the pET28a-*ChSPS1* expression vector by enzyme digestion. M: DNA Marker DL5000; 1: Positive control; 2: Enzyme digestion results.
(TIF)

S3 Fig Identification of the pBI121-*ChSPS1*expression vector by enzyme digestion. M: DNA Marker DL5000; 1: Positive control; 2: Enzyme digestion results.
(TIF)

S4 Fig Different culture phases of transgenic tobacco. (A, B, C) Differentiation stages of cultivation; (D, E, F) Rooting stages of cultivation; (G, H) Transplanting stages of cultivation.
(TIF)

S1 Text The sequencing data of *ChSPS1* gene. (DOCX)
