# Supplementary figures and images for: Cloning and characterization of the Cerasus humilis sucrose phosphate synthase gene (ChSPS1)
Source: PLoS One. 2017 Oct 16;12(10):e0186650. doi: 10.1371/journal.pone.0186650 (PMC5643142; doi:10.1371/journal.pone.0186650)

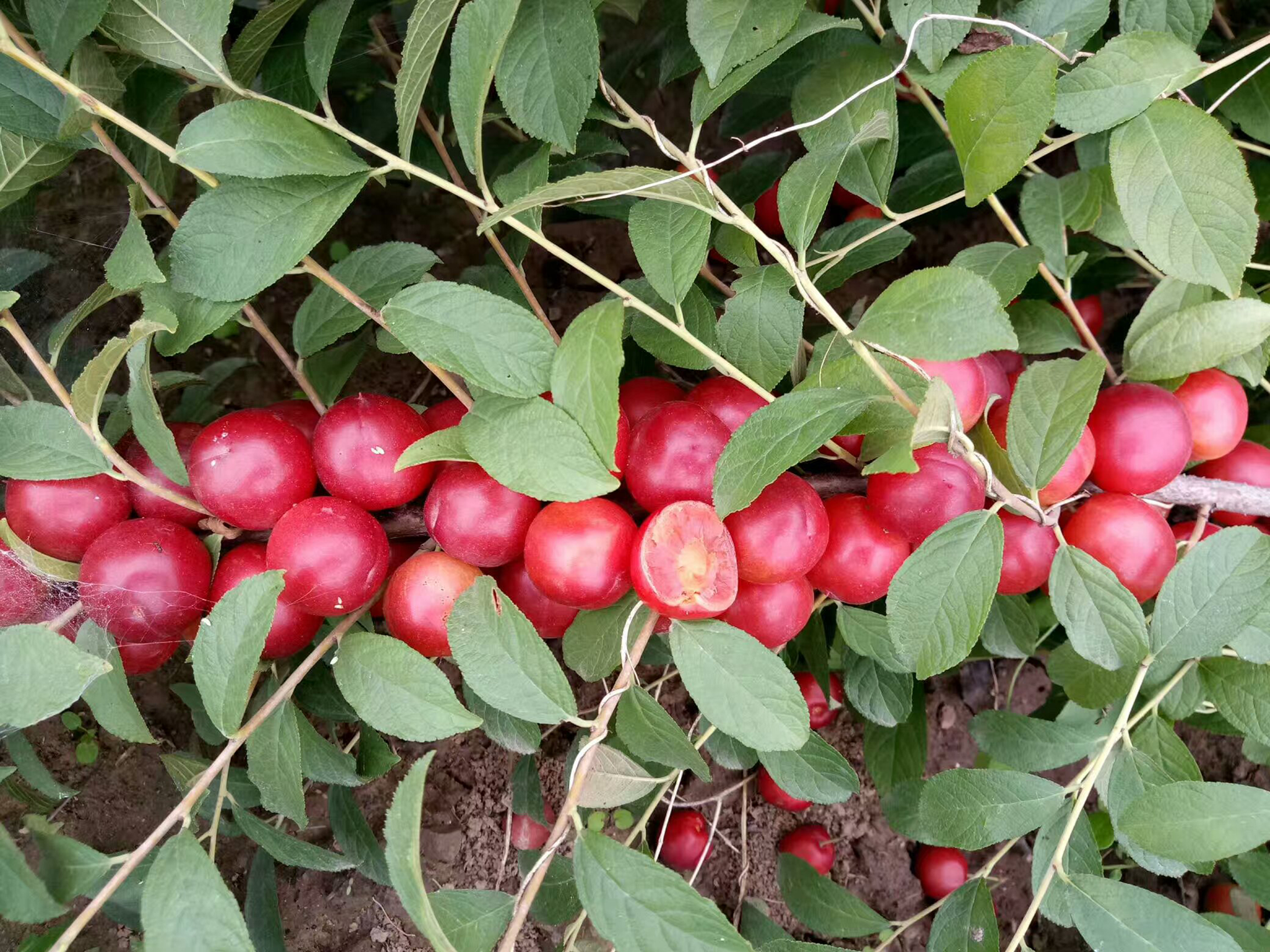

Supplement: S1 Fig — (TIF) [file pone.0186650.s001.tif]

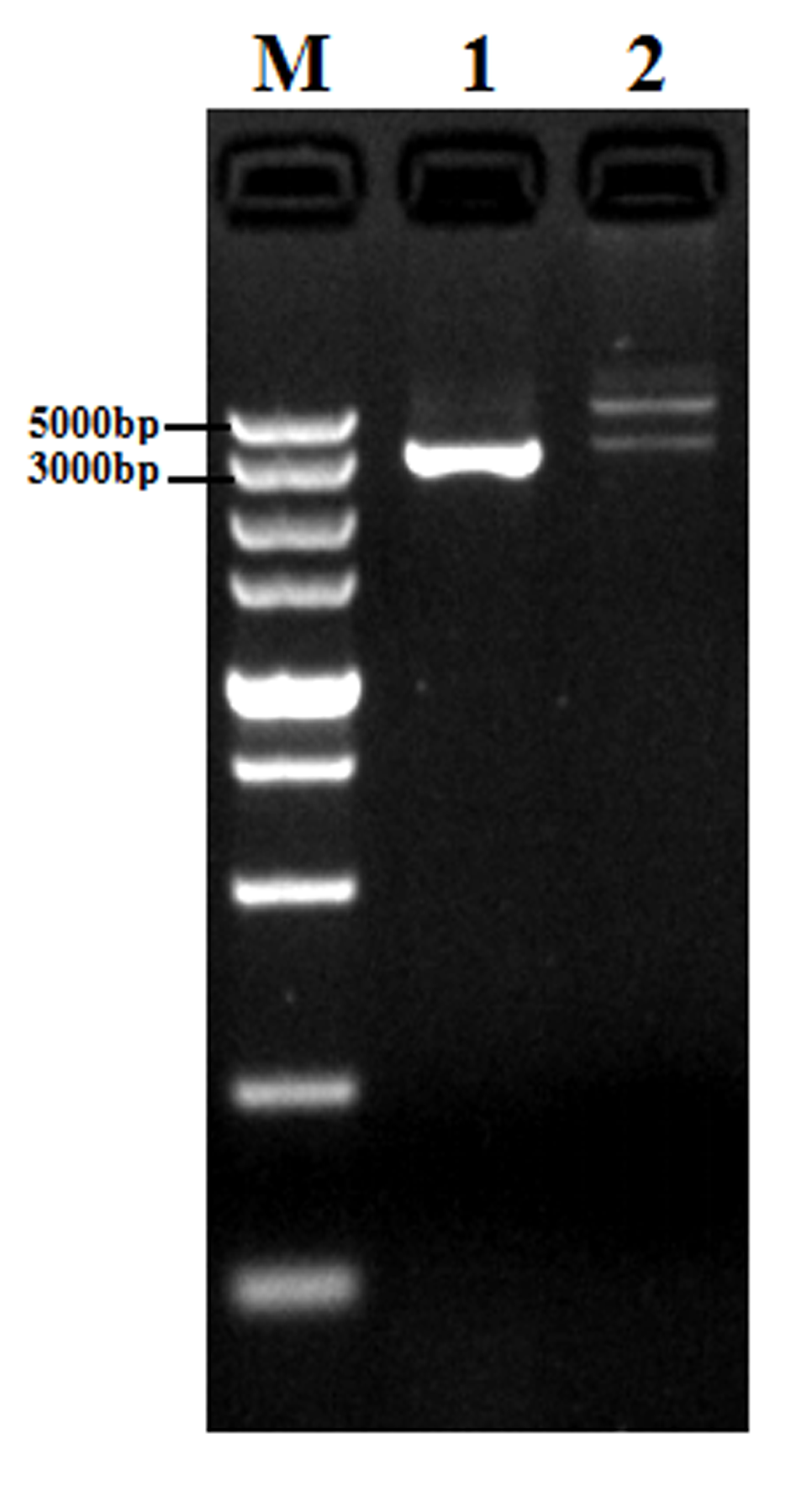

Supplement: S2 Fig — M: DNA Marker DL5000; 1: Positive control; 2: Enzyme digestion results. (TIF) [file pone.0186650.s002.tif]

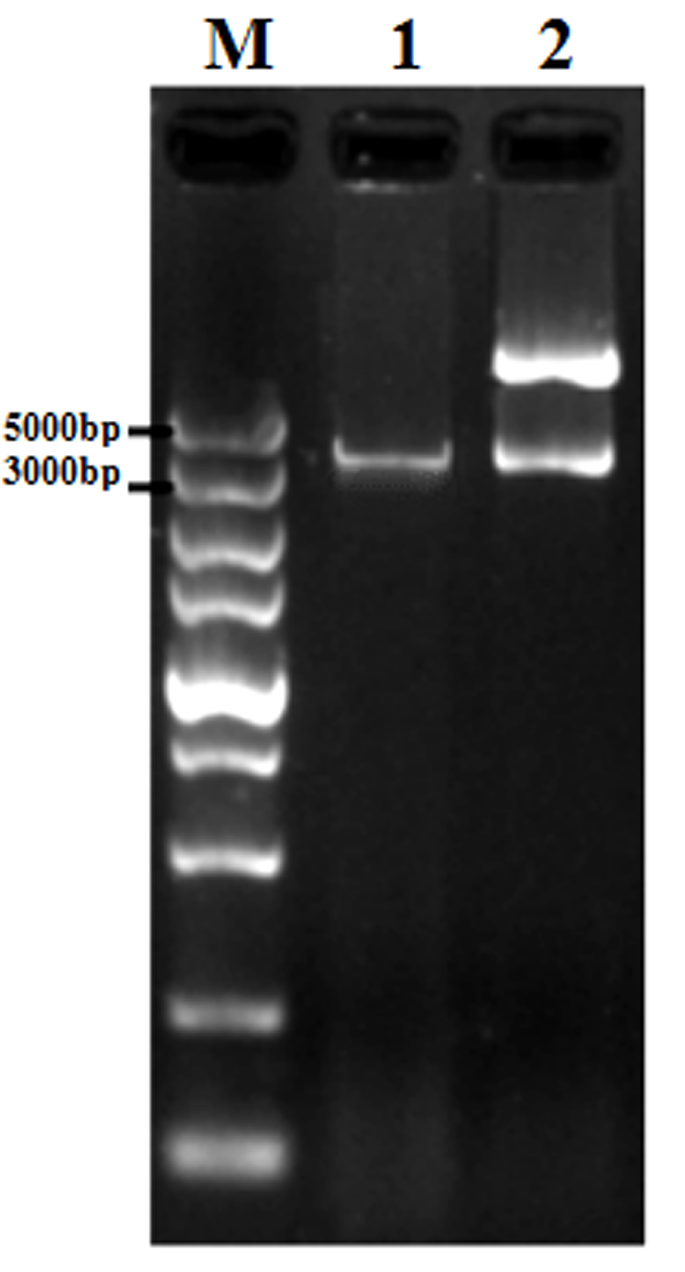

Supplement: S3 Fig — M: DNA Marker DL5000; 1: Positive control; 2: Enzyme digestion results. (TIF) [file pone.0186650.s003.tif]

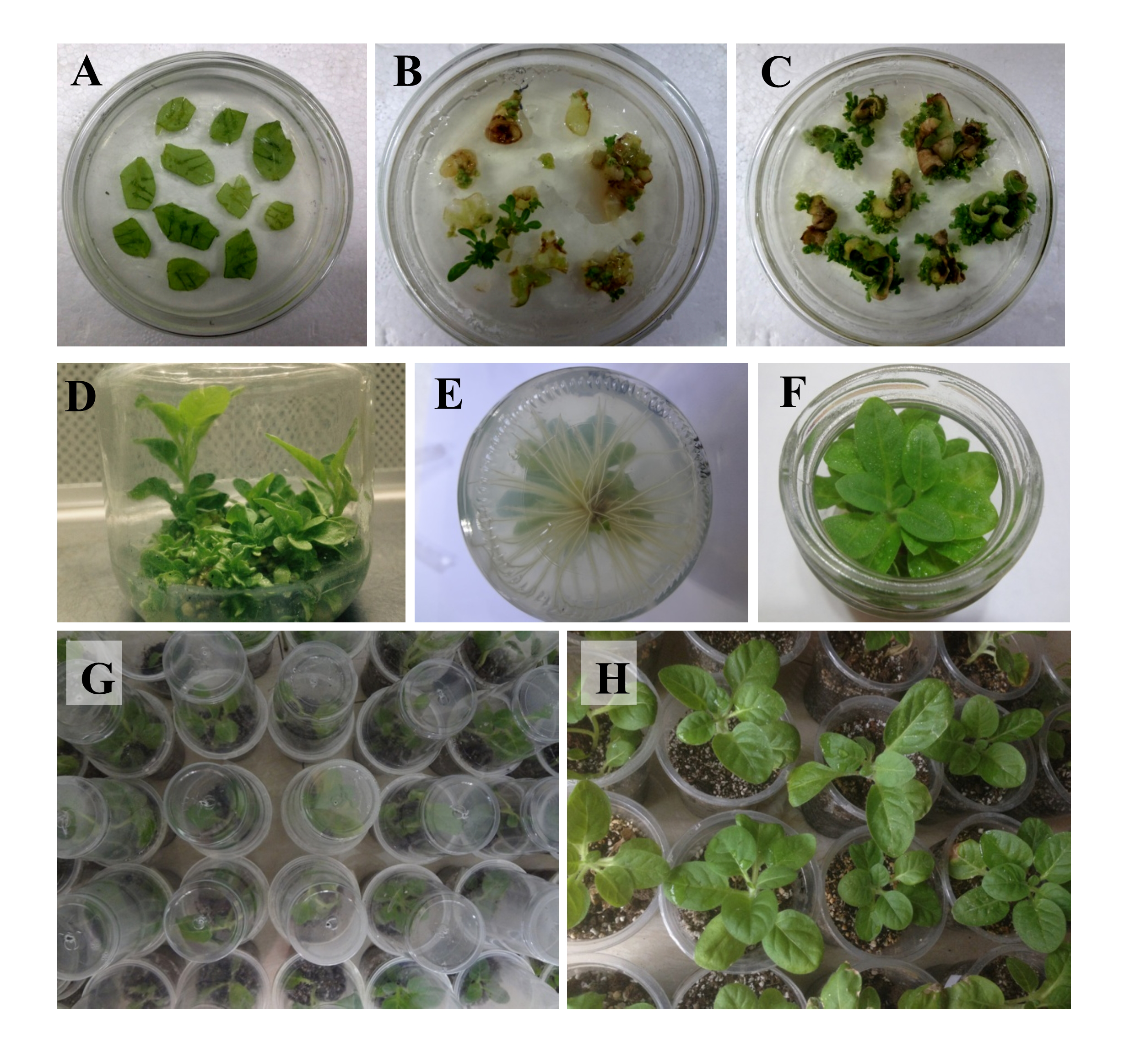

Supplement: S4 Fig — (A, B, C) Differentiation stages of cultivation; (D, E, F) Rooting stages of cultivation; (G, H) Transplanting stages of cultivation. (TIF) [file pone.0186650.s004.tif]
