# Supplementary material for: Cloning and characterization of the Cerasus humilis sucrose phosphate synthase gene (ChSPS1)
Source: PLoS One. 2017 Oct 16;12(10):e0186650. doi: 10.1371/journal.pone.0186650 (PMC5643142; doi:10.1371/journal.pone.0186650)
Supplement: S1 Text — (DOCX) [file pone.0186650.s005.docx]

Nucleotide sequences of *ChSPS1* in *C. humilis*

ATGGCGAGCAACGATTGGATAAACAGTTACCTGGAGGCGATTCTGGACGTGGGTCCAGGCCTCGTCGACGATGCCAAATCGTCCTTGCTGCTTAGAGAGAGAGGCCACTTCAGTCCCACCCGTTACTTTGTGGAGGAGGTCATCACCGGCTTCGATGAGACCGATCTCCACCGCTCTTGGGTTCGCGCTGCGGCGACGAGGAGCCCCCAGGAGAGGAACACCAGATTGGAGAATTTGTGCTGGCGGATTTGGAACTTAGCTCGCCAGAAGAAGCAGCTTGAGGGAGAGGAAGCTCAAAGGATGGCTAAACGTCGTATTGAACGTGAAAGAGGTCGCCGAGAAGCTACAGCTGATATGTCTGAAGACTTATCCGAGGGAGAGAAGGGAGATGTAGTGAGCGACATATCAGCTCATGGTGATAATAGCAGAGCCAGACTGCCTAGAATTAACTCCGTTGACACAATGGACATGTGGATTAGTCAGCAGAAGGGGAAAAAGCTATATATTGTACTAATAAGCCTTCATGGTCTTATACGAGGTGAAAATATGGAGCTCGGTCGTGACTCTGATACTGGTGGCCAGGTTAAGTATGTGGTAGAACTTGCACGGGCTTTGGGCACCACACCGGGAGTGTATAGAGTTGATCTGCTAACTAGACAAGTGTCATCACCGGATGTAGATTGGAGCTATGGTGAGCCCACAGAGATGCTGACTCCAATTAATGCAGACGGTTTTGCGGAAGAGATGGGGGAGAGCAGTGGTTCGTATATCATTCGTATACCATTCGGTCCCAAAGATAAATACATTCCTAAGGAAGAACTCTGGCCACACATCCCTGAATTTGTTGATGGTGCACTGAACCACATAATACAGATGTCCAAAGTTTTGGGTGAACAAGTTGGTGGTGGGAAGCCAGTCTGGCCTGTTGCCATCCATGGACATTATGCAGATGCTGGTGACTCTGCTGCCCTTTTATCTGGTGCTTTAAATGTCCCGATGCTTTTTACTGGCCACTCACTTGGCCGAGATAAGTTGGAGCAACTTTTAAAACAAGGCCGTCTTTCAAGGGATGAAATAAACACAACATACAAAATTATGCGTCGGATAGAGGCTGAGGAGTTGGCCCTTGATTCCTCTGAAATAGTTATTACCAGCACTAGACAGGAGATAGAAGAGCAATGGCGCTTGTATGATGGTTTTGATCCAATACTGGAGCGTAAACTGCGAGCTAGGATCAGGCGTAATGTCAGCTGTTATGGCAGGTTCATGCCCCGCATGGTGATAATTCCTCCTGGAATGGAGTTCCATCATATTGTTCCGCATGACGGTGATATGGATGGTGAGACAGAAGCAAATGAAGACCATCCTACTTCTCCAGACCCACCTATTTGGTCTGAGATAATGCGCTTCTTTACCAATCCTCGCAAGCCTATGATTCTTGCCCTTGCTAGACCAGATCCAAAAAAGAACATCACAACTTTGGTCAAAGCATTTGGAGAATGTCGCCCACTAAGAGAGCTTGCGAATCTTACACTAATTATGGGCAACCGCGATGGAATTGATGAAATGTCAGGCACAAGTTCTTCTGTTCTTCTCTCAGTACTCAAGCTTATTGACAAACATGATTTGTATGGGCAAGTGGCATACCCCAAACACCACAAACAGTCTGATGTTCCTGAGATTTATCGTCTAGCAGCAAAGACAAAGGGTGTTTTCATTAATCCAGCTTTCATTGAGCCGTTTGGACTCACATTAATTGAGGCAGCTGCTCATGGTTTACCTATTGTTGCCACAAAAAATGGGGGTCCTGTTGATATTCATCAGGTACTTGACAATGGTCTTCTTGTGGATCCTCATGATCAGCAGTCTATTGCTGATGCTCTCCTGAAGCTTGTTGCAGATAAGCAGCTCTGGGCAAGATGTCGGCAGAATGGGTTGAAGAACATTCACCTATTTTCTTGGCCTGAGCACTGCAAAACTTACCTGTCTCGAATAGCCAGTTGCAAACCAAGGCATCCACAATGGCAGAGAAGTGAGGATGGAGCAGAAACTACAGAATCAGATTCCCCAAGTGATTCCTTGAGAGATATACAGGATTTATCTTTGAACCTGAAGTTTTCATTGGATGGAGAAAAGAGCGGAACAAGTGTGAATGACAGTTCTTCAGAATATGAAGGAAATACTGCTGATAGAAGGAATAAAATAGAGAATGCTGTTTTGGCATGGTCAAAGGGTATTTCAAGGGACACACGGAAGGCTGGGTTCTCAGAGAAAGCAGACCATAACAGTGCTGGTAAGTTCCCAGTACTGAGAAGGAGGAAACATCTTCTTGTCATTGCTGTGGATTGTGATACCATTACGGATCTTATTGAAACTACAAGGAAGATTTTTGAGGCCACAGGAAAGGAAAGGACTGAAGGCTCTGTGGGGTTCATATTGTCAACATCCTTGACGATATCCGAGATAAGCTCATTTCTGGTCTCAGGGGGCTTGAGCCCCAATGACTTTGATGCATTTATTTGCAACAGTGGCAGTGATCTCTACTATCCATCTATTCATTCAGAGGAGCGTCCCTTTGTAGTTGACTTTTATTACCACTCACACATCGAATATCGTTGGGGTGGAGAAGGATTGAGGAAGACATTAGTACGTTGGGCAGGTTCAGTTAATGATAAGAAGACTGGGAGTGAGGAACAGATTGTTACTGCGGCTGATCAACTTTCAACTGACTATTGTTATGCTTTTAAAGTGCAGAAGCCTGGAAAGGTTCCCCCAGTGAAGGAGCTTCGGAAGTTGCTGAGGATTCAGGCTTTGCGGTGCCATGTAATTTACTGCCAAAATGGGACCAGAGTGAATGTAATTCCAGTATTGGCATCTCGTTCCCAAGCCCTCAGGTATTTATATCTTCGATGGGGTGTAGACTTATCAAAAGTGGTGGTTCTTGCTGGAGAATGCGGGGATACAGACTATGAAGGATTGCTTGGTGGGCTGCACAAAAGTGTAGTACTCAAGGGAGTTGCGAGCAATGCAATCAGTCAACTCCATACGAACCGAAACTACCCTCTATCAGATGTCCTGGCTCTTGACAGTCCCAACATTGTTCAGACCAGTGAAGGCTGTGGCAGCGACGATATCCGGGTTTCGTTGGAGAAACTCGGAGTTGTCAAGACGTAG

Nucleotide and deduced amino acid sequences of *ChSPS1* in *C. humilis*

1 ATGGCGAGCAACGATTGGATAAACAGTTACCTGGAGGCGATTCTGGACGTGGGTCCAGGCCTCGTCGACGATGCCAAATCGTCCTTGCTGCTTAGAGAGAGAGGC

1 M A S N D W I N S Y L E A I L D V G P G L V D D A K S S L L L R E R G

106 CACTTCAGTCCCACCCGTTACTTTGTGGAGGAAGTCATCACCGGCTTCGATGAGACCGATCTCCACCGCTCTTGGGTTCGCGCTGCGGCGACGAGGAGCCCCCAG

36 H F S P T R Y F V E E V I T G F D E T D L H R S W V R A A A T R S P Q

211 GAGAGGAACACCAGATTGGAGAATTTGTGCTGGCGGATTTGGAACTTAGCTCGCCAGAAGAAGCAGCTTGAGGGAGAGGAAGCTCAAAGGATGGCTAAACGTCGT

71 E R N T R L E N L C W R I W N L A R Q K K Q L E G E E A Q R M A K R R

316 ATTGAACGTGAAAGAGGTCGCCGAGAAGCTACAGCTGATATGTCTGAAGACTTATCCGAGGGAGAGAAGGGAGATGTAGTGAGCGACATATCAGCTCATGGTGAT

106 I E R E R G R R E A T A D M S E D L S E G E K G D V V S D I S A H G D

421 AATAGCAGAGCCAGACTGCCTAGAATTAACTCCGTTGACACAATGGACATGTGGATTAGTCAGCAGAAGGGGAAAAAGCTATATATTGTACTAATAAGCTTTCAT

141 N S R A R L P R I N S V D T M D M W I S Q Q K G K K L Y I V L I S F H

526 GGTCTTATACGAGGTGAAAATATGGAGCTCGGTCGTGACTCTGATACTGGTGGCCAGGTTAAGTATGTGGTGGAACTTGCACGGGCTCTGGGCACCACACCGGGA

176 G L I R G E N M E L G R D S D T G G Q V K Y V V E L A R A L G T T P G

631 GTGTATAGAGTTGATCTGCTAACTAGACAAGTGTCATCACCGGATGTAGATTGGAGCTATGGTGAGCCCACAGAGATGCTGACTCCAATTAATGCAGACGGTTTT

211 V Y R V D L L T R Q V S S P D V D W S Y G E P T E M L T P I N A D G F

736 GCGGAAGAGATGGGGGAGAGCAGTGGTTCGTATATCATTCGTATACCATTCGGTCCCAAAGATAAATACATTCCTAAGGAAGAACTCTGGCCACACATCCCTGAA

246 A E E M G E S S G S Y I I R I P F G P K D K Y I P K E E L W P H I P E

841 TTTGTTGATGGTGCACTGAACCACATAATACAGATGTCCAAAGTTTTGGGTGAACAAGTTGGTGGTGGGAAGCCAGTCTGGCCTGTTGCCATCCATGGACATTAT

281 F V D G A L N H I I Q M S K V L G E Q V G G G K P V W P V A I H G H Y

946 GCAGATGCTGGTGACTCTGCTGCCCTTTTATCTGGTGCTTTAAATGTCCCGATGCTTTTTACTGGCCACTCACTCGGCCGAGATAAGTTGGAGCAACTTTTAAAA

316 A D A G D S A A L L S G A L N V P M L F T G H S L G R D K L E Q L L K

1051 CAAGGCCGTCTTTCAAGGGATGAAATAAACACAACATACAAAATTATGCGTCGGATAGAGGCTGAGGAGTTGGCCCTTGATTCCTCTGAAATAGTTATTACCAGC

351 Q G R L S R D E I N T T Y K I M R R I E A E E L A L D S S E I V I T S

1156 ACTAGACAGGAGATAGAAGAGCAATGGCGCTTGTATGATGGTTTTGATCCAATACTGGAGCGTAAACTGCGAGCTAGGATCAGGCGTAATGTCAGCTGTTATGGC

386 T R Q E I E E Q W R L Y D G F D P I L E R K L R A R I R R N V S C Y G

1261 AGGTTCATGCCCCGCATGGTGATAATTCCTCCTGGAATGGAGTTCCATCATATTGTTCCGCATGACGGTGATATGGATGGTGAGACAGAAGCAAATGAAGACCAT

421 R F M P R M V I I P P G M E F H H I V P H D G D M D G E T E A N E D H

1366 CCTACTTCTCCAGACCCACCTATTTGGTCTGAGATAATGCGCTTCTTTACCAATCCTCGCAAGCCTATGATTCTTGCCCTTGCTAGACCAGATCCAAAAAAGAAC

456 P T S P D P P I W S E I M R F F T N P R K P M I L A L A R P D P K K N

1471 ATCACAACTTTGGTCAAAGCATTTGGAGAATGTCGCCCACTAAGAGAGCTTGCGAATCTTACACTAATTATGGGCAACCGCGATGGAATTGATGAAATGTCAGGC

491 I T T L V K A F G E C R P L R E L A N L T L I M G N R D G I D E M S G

1576 ACAAGTTCTTCTGTTCTTCTCTCAGTACTCAAGCTTATTGACAAACATGATTTGTATGGGCAAGTGGCATACCCCAAACACCACAAACAGTCTGATGTTCCTGAG

526 T S S S V L L S V L K L I D K H D L Y G Q V A Y P K H H K Q S D V P E

1681 ATTTATCGTCTAGCAGCAAAGACAAAGGGTGTTTTCATTAATCCAGCTTTCATTGAGCCGTTTGGACTCACGTTAATTGAGGCAGCTGCTCATGGTTTACCTATT

561 I Y R L A A K T K G V F I N P A F I E P F G L T L I E A A A H G L P I

1786 GTTGCCACAAAAAATGGGGGTCCTGTTGATATTCATCAGGTACTTGACAATGGTCTTCTTGTGGATCCTCATGATCAGCAGTCTATTGCTGATGCTCTCCTGAAG

596 V A T K N G G P V D I H Q V L D N G L L V D P H D Q Q S I A D A L L K

1891 CTTGTTGCAGATAAGCAGCTCTGGGCAAGATGTCGGCAGAATGGGTTGAAGAACATTCACCTATTTTCTTGGCCTGAGCACTGCAAAACTTACCTGTCTCGAATA

631 L V A D K Q L W A R C R Q N G L K N I H L F S W P E H C K T Y L S R I

1996 GCCAGTTGCAAACCAAGGCATCCACAATGGCAGAGAAGTGAGGATGGAGCAGAAACTACAGAATCAGATTCCCCAAGTGATTCCTTGAGAGATATACAGGATTTA

666 A S C K P R H P Q W Q R S E D G A E T T E S D S P S D S L R D I Q D L

2101 TCTTTGAACCTGAAGTTTTCATTGGATGGAGAAAAGAGCGGAACAAGTGTGAATGACAGTTCTTCAGAATATGAAGGAAATACTGCTGATAGAAGGAATAAAATA

701 S L N L K F S L D G E K S G T S V N D S S S E Y E G N T A D R R N K I

2206 GAGAATGCTGTTTTGGCATGGTCAAAGGGTATTTCAAGGGACACACGGAAGGCTGGGCTCTCAGAGAAAGCAGACCATAACAGTGCTGGTAAGTTCCCAGTACTG

736 E N A V L A W S K G I S R D T R K A G L S E K A D H N S A G K F P V L

2311 AGAAGGAGGAAACATCTTCTTGTCATTGCTGTGGATTGTGATACCATTACAGATCTTATTGAAACTACAAGGAAGATTTTTGAGGCCACAGGAAAGGAAAGGACT

771 R R R K H L L V I A V D C D T I T D L I E T T R K I F E A T G K E R T

2416 GAAGGCTCTGTGGGGTTCATATTGTCAACATCCTTGGCGATATCCGAGATAAGCTCATTTCTGGTCTCAGGGGGCTTGAGCCCCAATGACTTTGATGCATTTATT

806 E G S V G F I L S T S L A I S E I S S F L V S G G L S P N D F D A F I

2521 TGCAACAGTGGCAGTGATCTCTACTATCCATCTATTCATTCAGAGGAGCGTCCCTTTGTAGTTGACTTTTATTACCACTCACACATCGAATATCGTTGGGGTGGA

841 C N S G S D L Y Y P S I H S E E R P F V V D F Y Y H S H I E Y R W G G

2626 GAAGGATTGAGGAAGACATTAGTACGTTGGGCAGGTTCAGTTAATGATAAGAAGACTGGGAGTGAGGAACAGATTGTTACTGCGGCTGATCAACTTTCAACTGAC

876 E G L R K T L V R W A G S V N D K K T G S E E Q I V T A A D Q L S T D

273 TATTGTTATGCTTTTAAAGTGCAGAAGCCTGGAAAGGTTCCCCCAGTGAAGGAGCTTCGGAAGTTGCTGAGGATTCAGGCTTTGCGGTGCCATGTAATTTACTGC

911 Y C Y A F K V Q K P G K V P P V K E L R K L L R I Q A L R C H V I Y C

2836 CAAAATGGGACCAGAGTGAATGTAATTCCAGTATTGGCATCTCGTTCCCAAGCCCTCAGGTATTTATATCTTCGATGGGGTGTAGACTTATCAAAAGTGGTGGTT

946 Q N G T R V N V I P V L A S R S Q A L R Y L Y L R W G V D L S K V V V

2941 CTTGCTGGAGAATGCGGGGATACAGACTATGAAGGATTGCTTGGTGGGCTGCACAAAAGTGTAGTACTCAAGGGAGTTGCGAGCAATGCAATCAGTCAACTCCAT

981 L A G E C G D T D Y E G L L G G L H K S V V L K G V A S N A I S Q L H

3046 ACGAACCGAGACTACCCTCTATCAGATGTCCTGGCTCTTGACAGTCCCAACATTGTTCAGACCAGTGAAGGCTGTGGCAGCGACGATATCCGGGTTTCGTTGGAG

1016 T N R D Y P L S D V L A L D S P N I V Q T S E G C G S D D I R V S L E

3151 AAACTCGGAGTTGTCAAGACGTAG

1051 K L G V V K T *
